# The impact of COVID-19 on the male reproductive tract and fertility: A systematic review

**DOI:** 10.1080/2090598X.2021.1955554

**Published:** 2021-08-09

**Authors:** Pallav Sengupta, Kristian Leisegang, Ashok Agarwal

**Affiliations:** a Faculty of Medicine, Bioscience and Nursing, MAHSA University, Malaysia; b School of Natural Medicine, Faculty of Community and Health Sciences, University of the Western Cape, Bellville, South Africa; c American Center for Reproductive Medicine, Cleveland Clinic, Cleveland, OH, USA

**Keywords:** SARS-CoV-2, COVID-19, male infertility, semen parameters, orchitis, testicular histology, testosterone

## Abstract

**Objective:**

The COVID-19 pandemic, caused by the acute respiratory syndrome-coronavirus 2 (SARS-CoV-2), remains an ongoing public health challenge. Although males are affected slightly more than females, the impact of SARS-COV-2 on male reproductive system remains unclear. This systematic review aims to provide a concise update on the effects of COVID-19 on male reproductive health, including the presence of viral RNA in semen, and the impact on semen quality, testicular histology, testicular pain and male reproductive hormones. The global health is fronting an immediate as well as impending threat from the novel coronavirus (SARS-CoV-2) causing coronavirus disease (COVID-19), that inflicts more males than females. Evidence suggest that male reproductive system is susceptible to this viral infection. However, there are still several pertinent queries that remain to be fully explained regarding the mechanism in testicular SARS-CoV-2 dynamics and the exact mode of its actions. Thus, the present systematic review aims to provide a concise update on the effects of coronavirus disease 2019 (COVID-19) on male reproduction..

**Methods:**

A systematic review was conducted according to Preferred Reporting Items for Systematic Reviews and Meta-Analyses guidelines searching the PubMed database. Eligibility for inclusion were original human studies evaluating the impact of COVID-19 on male reproductive health. Specific outcomes required for inclusion were at least one of the following: i) seminal detection of mRNA virus, or evaluation of ii) semen analysis, iii) testicular histology or ultrasonography, iv) testicular clinical symptoms and/or v) male reproductive hormones in COVID-19-positive patients.

**Results:**

Of 553 retrieved articles, 25 met the inclusion criteria. This included studies primarily investigating the presence of viral RNA in semen (*n* = 12), semen quality (*n* = 2), testicular histology (*n* = 5), testicular pain (*n* = 2) and male reproductive hormones (*n*= 4). Results show little evidence for the presence of viral RNA in semen, although COVID-19 seems to affect seminal parameters, induce orchitis, and cause hypogonadism. Mortality cases suggest severe histological disruption of testicular architecture, probably due to a systemic and local reproductive tract inflammatory response and oxidative stress-induced damage.

**Conclusions:**

Clinical evaluation of the male reproductive tract, seminal parameters and reproductive hormones is recommended in patients with current or a history of COVID-19, particularly in males undergoing fertility treatment. Any long-term negative impact on male reproduction remains unexplored and an important future consideration.

## Introduction

The coronavirus disease 2019 (COVID‑19) is caused by the severe acute respiratory syndrome-coronavirus-2 (SARS-CoV-2) [1,2], which is a highly transmissible coronavirus (CoV) first identified in 2019. COVID-19 has subsequently emerged as a global pandemic and is a significant threat to public health and safety [2]. On the 17 March 2021, >120 million confirmed cases were reported, along with >2.5 million deaths globally [3]. As part of the CoV family, SARS-CoV-2 is genetically 50% similar to Middle East Respiratory Syndrome (MERS)-CoV, 79.5% similar to SARS-CoV-1, and 96.5% similar to Bat-CoV [4–6]. However, due to its high transmission rate, SARS-CoV-2 is considered a more significant public health threat compared to SARS-CoV-1 and MERS-CoV [6].

The SARS-CoV-2 virus gains access to human cells through binding to cellular angiotensin-converting enzyme 2 (ACE2) receptors via the receptor-binding domain as part of the spike (S) protein. This further requires priming by cellular transmembrane protease, serine 2 (TMPRSS2) [7,8]. Following infection through exposure to respiratory droplets, the incubation period is usually 3–7 days [8]. The most frequent symptoms reported include fever (>75%), cough (>60%), fatigue (>25%), dyspnoea (>20%) and sputum production (>18.0%). Other less frequent but common symptoms include anorexia, myalgia, pharyngitis, rhinitis and diarrhoea [9–11]. COVID-19 pathophysiology is considered a two-phase disease. The first phase is characterised by increased viral transmission rates and replication, particularly through the respiratory tract due to the high expression of ACE2 and TMPRSS2. Phase 2 is a host-specific (including gender- and age-specific) characterised by a hyperinflammatory response, hypercytokinaemia and collateral tissue damage that may result in acute respiratory distress and systemic failure [7,8]. Increased risk for phase 2 complications includes older age, obesity, hypertension, diabetes and chronic respiratory disease [12].

Although epidemiological data suggests that men may have an increased risk of COVID-19-related morbidity and mortality [13–19], the potential impact on male reproduction and any mechanisms remain poorly understood. Numerous review articles have postulated the potential of SARS-CoV-2 to infect male reproductive tissues due to the presence of ACE2 receptors. This has led to the hypothesis that SARS-CoV-2 may gain access to the reproductive tract, may be sexually transmitted, and that COVID-19 may directly or indirectly impair reproductive function and/or disrupt the hypothalamus-pituitary-testis axis [20–25].

As COVID-19 remains an ongoing public health challenge, there is an increasing number of studies investigating its impact on testicular functions and reproductive hormones. Therefore, the present systematic review aims to provide a concise update on the effects of COVID-19 on male reproductive health. This will focus on presence of viral RNA in semen, semen quality, testicular histology, testicular pain and male reproductive hormones.

## Methods

The literature search was conducted based on the Preferred Reporting Items for Systematic Reviews and Meta-Analyses (PRISMA) guidelines [26]. The PubMed database was searched (last updated search on 7 February 2021) using the following keyword string adapted from Khalili et al. (2020) [27]: (‘severe acute respiratory syndrome–coronavirus 2’ OR ‘severe acute respiratory syndrome coronavirus 2’ OR ‘COVID-19’ OR ‘SARS-CoV-2’ OR ‘SARS CoV2’ OR ‘SARS CoV 2’) AND (‘semen’ OR ‘sperm*’ OR ‘seminal’ OR ‘testes’ OR ‘testicular’ OR ‘male fertil*’ OR ‘male infertil*’ OR ‘epididymis’ OR ‘prostate’ OR ‘testosterone’ OR ‘LH’ OR ‘FSH’ OR ‘cryopreservation’). Inclusion criteria were original human studies evaluating the impact of COVID-19 on male reproductive health. Specific outcomes required for inclusion were at least one of the following: i) seminal detection of mRNA virus, ii) semen analysis, iii) testicular histology or ultrasonography (US), iv) testicular clinical symptoms, and/or v) male reproductive hormones (testosterone, LH and/or FSH). Exclusion criteria were narrative or systematic reviews, and meta-analysis, pre-clinical studies and non-English studies. Retrieved articles were manually screened based on the titles and abstracts, and subsequently verified for eligibility by two authors (P.S. and K.L.), and any disputes were settled by a third author (A.A.).

## Results

**Figure 1.**
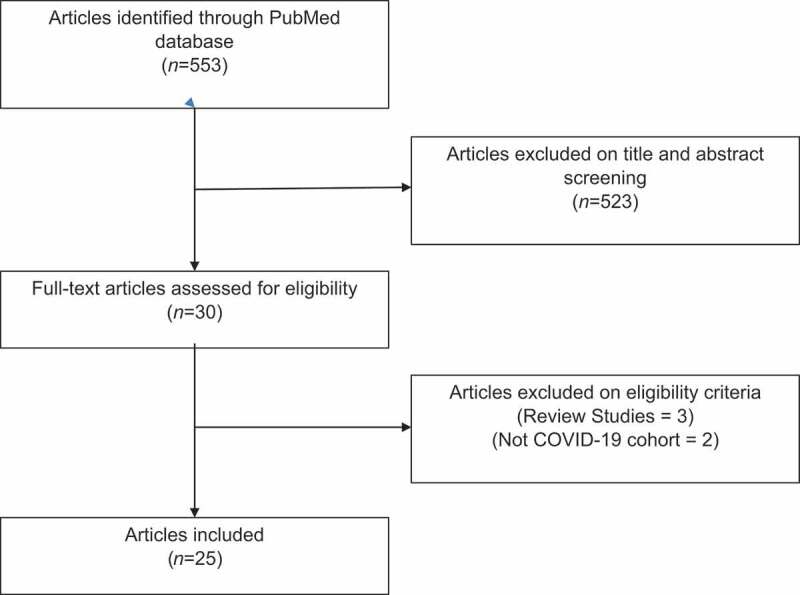
PRISMA flow diagram of systematic search

A total of 553 articles were retrieved following the PubMed database extraction. Of these, 523 articles were removed following screening of titles and abstracts. A total of 30 full-text articles were assessed for eligibility, with five articles removed as they were review articles (three) or not a COVID-19 cohort (two). Therefore, a total of 25 articles were included into this review (Figure 1). These were further classified based on the primary objective of the study: presence of viral RNA in semen (12) (Table 1), semen quality (two) (Table 2), testicular histology (five) (Table 3), testicular pain (two) (Table 4), and male reproductive hormones (four) (Table 5)

**Table 1.** Available literature investigating SARS-CoV-2 RNA detected in semen

Reference	Study design	Cohort (*n*)	Control (*n*)	Virus in semen	Additional findings
^28^	Cross-sectional cohort study	Patients recovering from COVID-19 (23); Patients with acute stage of COVID-19 infection (15)	–	SARS-CoV-2 RNA detected in semen from 6 patients (4 patients with acute infection and 2 recovering patients)	–
^29^	Cross-sectional cohort study	Sexually active men recovered from COVID-19 (43)	–	SARS-CoV-2 RNA detected in semen of one patient	25% found to be oligo-crypto-azoospermic that was related to COVID-19 severity; 76% found to have increased seminal IL-8
^32^	Cross-sectional cohort study	Patients with acute stage of COVID-19 infection (16)	–	No SARS-CoV-2 RNA detected in semen	–
^33^	Cross-sectional case-controlled study	Patients recovering from COVID-19 (74)	Age-matched healthy controls (174)	No SARS-CoV-2 RNA detected in semen or urine	Significantly reduced sperm concentration, total sperm count, and total motility compared controls
^34^	Cross-sectional cohort study	Patients recovering from COVID-19 (34)	–	No SARS-CoV-2 RNA detected in semen	Scrotal discomfort indicative of orchitis in 19% cases
^35^	Cross-sectional case-controlled study	COVID-19 pre-treatment (10); COVID-19 post-treatment (10)	Controls (10)	No SARS-CoV-2 RNA detected in semen	Reduced levels of serum FSH, LH and T have been found in COVID-19 group compared to controls; significant reduced sperm morphology in COVID-19 group compared to controls; no significant differences between groups after treatment.
^36^	Cross-sectional cohort study	Patients recovering from COVID-19 (13)	–	No SARS-CoV-2 RNA detected in semen	No SARS-CoV-2 RNA detected in testes on autopsy of a 67-year-old patient
^37^	Cross-sectional case-controlled study	Patients recovering from COVID-19 (16); Patients with acute stage of COVID-19 infection (2)	Patients with no antibodies (14)	No SARS-CoV-2 RNA detected in semen in any group	Moderate infection significantly impaired sperm quality (sperm concentration, progressive motility, total number of complete motility) compared with men recovered from a mild infection and the control group
^38^	Cross-sectional cohort study	Men diagnosed with COVID-19 (6)	–	No SARS-CoV-2 RNA detected in semen	–
^39^	Cross-sectional cohort study	COVID-19 patients with mild or no symptoms (9)	–	No SARS-CoV-2 RNA detected in semen	–
^40^	Cross-sectional cohort study	COVID-19 patients in acute and recovery phase (23)	–	No SARS-CoV-2 RNA detected in semen	Total sperm count, total motility, and sperm morphology within normal ranges
^41^	Case study	31-year-old man with mild COVID-19 (1)	–	No SARS-CoV-2 RNA was detected in semen and urine samples	–

**Table 2.** Available literature investigating SARS-CoV-2 infection on semen quality

Reference	Study design	Cohort (*n*)	Control (*n*)	Semen quality	Other findings
^43^	Cross-sectional case-controlled study	Recovering COVID-19 inpatients (23); autopsied testicular and epididymal specimens (6)	Age-matched controls for inpatients (23); age-matched controls for autopsies (6)	Decreased sperm concentration compared to controls; in the recovering patients, 39.1% had oligozoospermia and 60.9% had increased leucocytes; increased seminal levels of IL-6, TNF-α, and MCP-1 compared to control	Autopsied testicular and epididymal samples showed oedema, congestion and exudation of erythrocytes; thinning of seminiferous tubules and increased number of seminiferous apoptotic cells was observed in the autopsied testes; increased concentration of CD3^+^ and CD68^+^ in the interstitial cells of testicular tissue and the presence of IgG within seminiferous tubules of autopsied testes.
^44^	Case-controlled longitudinal study	COVID-19 recovering patients (84)	Healthy men (105)	Significant impairments in semen volume, sperm concentration progressive motility, sperm morphology compared to control; significantly higher levels of seminal plasma ACE2 enzymatic activity, IL-1β, IL-6, IL-8, IL-10, TGF-β, TNF-α, IFN-α, IFN-γ, ROS, caspase-8, caspase-9, and caspase-3 activity and lower levels of SOD activity compared to control group.	–

**Table 3.** Available literature investigating SARS-CoV-2 infection on testicular histology and/or ultrasonography

Reference	Study design	Cohort (*n*)	Control (*n*)	Histology	Other findings
^31^	Cross-sectional case-controlled study	COVID-19 patients (6)	Uninfected controls with similar comorbidities and age distribution (3)	Impaired spermatogenesis in 3 COVID-19-positive cases; viral spike protein particles observed in testis of one COVID-19-positive autopsy case, associated with infiltration of macrophages and leucocytes; reduced expression of ACE2 receptors in testes of COVID-19 patients with normal spermatogenesis compared to COVID-19 patients with impaired spermatogenesis.	–
^42^	Cross-sectional case-controlled study	Autopsies of males who died of COVID-19 (10)	Uninfected controls with similar comorbidities and age distribution (7)	Acute testicular injury reported that is related to oxidative stress (spermatocytes elongation and sloughing with Sertoli cell swelling) compared to chronic damage in controls (decreased spermatogenesis and Leydig cells)	No SARS-CoV-2 RNA detected in testes on autopsy
^45^	Cross-sectional case-controlled study	COVID-19 patients (5)	Uninfected controls (3)	Morphological disruptions of testes reported. GCs degeneration and sloughing in seminiferous tubule lumen in the COVID-19 patients.	–
^46^	Cross-sectional case-controlled study	Autopsy of testes of COVID-19 cases (11)	Uninfected controls (5)	Sertoli cell swelling, vacuolisation and detachment from basement membranes; significantly reduced Leydig cells compared to control; interstitial oedema and mild inflammatory infiltrates; no microscopy detection of viral particles	SARS-Cov-2 RNA detected in testes of 1 COVID-19 sample; spermatogenesis was not altered.
^47^	Retrospective cohort study	COVID-19 patients (142)	–	Orchitis, epididymitis, or epididymo‐orchitis in 22.5% of patients with COVID-19, associated with thickened tunica albuginea and increased vascular flow as common findings	–

**Table 4.** Available literature investigating SARS-CoV-2 infection on male reproductive clinical symptoms

Reference	Study design	Cohort (*n*)	Control (*n*)	Clinical Symptoms	Other findings
48	Cross-sectional case-controlled study	COVID-19 patients with testicular pain (10)	COVID-19 patients without testicular pain (81)	Testicular pain prevailed in 10.98% patients (*n*= 91), with one case of clinical epididymo‐orchitis reported	No difference for neutrophil count, lymphocyte count, CRP, D-dimers or duration of COVID-19 infection between groups
49	Case study	42-year-old-male patient (1)	–	Presented with abdominal and testicular pain without respiratory symptoms	–

**Table 5.** Available literature investigating SARS-CoV-2 infection on male reproductive hormones

Reference	Study design	Cohort (*n*)	Control (*n*)	Hormones	Other findings
^30^	Cross-sectional case-controlled study	Hospitalised COVID-19 patients (119)	Age-matched control (273)	Decreased testosterone and increased LH; decreased testosterone:LH ratio that had a negative association with WBCs and levels of CRP	Reduced sperm concentration and increased SDF in 33.3% of 11 cases; SARS-CoV-2 RNA detected in semen from 1 patient (mild COVID-19), but not from 11 moderate COVID-19 infections
^50^	Cross-sectional case-controlled study	COVID-19 patients (89)	Non–COVID-19 respiratory tract infection (30); Controls (143)	Decreased testosterone and increased LH and prolactin with no change for FSH	Significantly lower WBC and lymphocyte count compared to non-Covid-19 and control patients; CRP was significantly higher in COVID-19 and non-COVID-19 patients compared to control
^51^	Cross-sectional case-controlled study	COVID‐19 outpatients (24)	Outpatients negative for COVID-19 (20)	No difference for testosterone, LH or FSH between positive and negative COVID-19 patients; decreased testosterone in patients with COVID-19 pneumonia compared to COVID-19 without pneumonia	–
^52^	Cross-sectional case-controlled study	SARS‐CoV‐2 pneumonia who worsened or died (4)	SARS‐CoV‐2 pneumonia who remained stable (6) or improved (21)	Decreased total and free testosterone in patients who had worsened or died compared to patients who recovered or remined stable	Significantly higher neutrophils, potassium, CRP, procalcitonin, LDH and lower lymphocytes in patients who had worsened or died compared to patients who recovered or remined stable

LDH, lactate dehydrogenase.

## SARS-CoV-2 in human semen

The results of this review retrieved 12 studies that primarily investigated the presence of SARS-CoV-2 RNA in human seminal fluid, with only two studies detecting the presence of the virus in semen (Table 1).

In 38 male patients (aged >15 years) that tested positive for COVID-19 at Shangqiu Municipal Hospital, SARS-CoV-2 RNA was detected in the semen samples of four acute stage patients and two recovery phase patients [28]. Gacci et al. [29] (2021) reported only one patient in a cohort of 43 sexually active men recovered from COVID-19 to have evidence of SARS-CoV-2 RNA in seminal fluid, who subsequently tested negative on a nasopharyngeal test the next day. In an additional study that included a sub-analysis investigating the impact of COVID-19 on semen analysis in 12 SAR2-CoV-2-positive patients, Ma et al. [30] (2021) reported the presence of the viral RNA in one case of mild infection, with the remaining 11 cases (moderate infection) being negative. Achua et al. [31] (2021) reported testicular viral spike protein particles in one out of four COVID-19-positive case autopsies on microscopy, and from a sample obtained from one alive positive COVID-19 patient.

The remaining studies did not detect the virus in seminal fluid. Kayaaslan et al. [32] (2020) investigated semen of 16 hospitalised patients (median age 33.5 years) with SARS-CoV-2 infection confirmed on nasopharyngeal swabs in a cross-sectional cohort study, with 11 semen samples collected within 1 day of a positive diagnosis and all samples within 7 days. In these acutely ill patients, no viral RNA was detected in the semen. Similarly, no viral RNA detection in seminal fluid was reported in a case-controlled study of 74 men (median age 34 years) who had been hospitalised and confirmed positive for COVID-19 and subsequently were recovering. The authors reported that 14.9% were asymptomatic (mild), 41.9% were classified as moderate, and 43.2% had severe pneumonia [33]. In 34 Chinese male patients (median age 37 years) recovering from COVID-19, the virus was not detected at a median of 31 days after a confirmed nasopharyngeal diagnosis [34]. Another case-controlled cross-sectional study that investigated confirmed and probable COVID-19 cases before (10) and after treatment (10) compared to control (10) (mean [SD] age for entire cohort 37.2 [8.6] years) also found no evidence of SARS-CoV-2 RNA in seminal fluid [35]. Similarly, Song et al. [36] (2020) reported no viral RNA in semen of 12 men (aged 22–38 years) recovering from COVID-19 (two negative tests following a positive diagnosis) in Wuhan, China, where 11 patients had mild-to-moderate symptoms and one was asymptomatic. In a case-controlled cohort study that included 16 patients recovering from COVID-19 (mean [SD] age 42.2 [9.9] years), two patients with acute COVID-19 and 14 control patients (mean [SD] age 33.4 [13.1] years) that were negative for SARS-CoV-2, no viral RNA was found in seminal fluid in any participant [37]. A small cohort of six men (aged 28–45 years) that were diagnosed with COVID-19 based on symptoms and a positive nasopharyngeal swab were negative for seminal viral RNA, while still positive for saliva and nasal swab viral RNA [38]. In a cohort of nine Italian men (median age 42 years) positive for nasopharyngeal swab and a diagnosis of mild (eight) or asymptomatic (one) COVID-19 that did not require hospitalisation, none of the semen samples showed the presence of viral RNA [39]. Similarly, no viral RNA was identified in seminal fluid of 23 men (age 20–62 years) within the acute phase or convalescence period of COVID-19. The median time from diagnosis to the semen sample was 32 days. Among these 23 men, the virus had been cleared in 11 of them at time of semen analysis, with 12 men having positive viral RNA in sputum or faecal specimens [40]. This is supported by a case study by Paoli et al. [41] (2020) on a 31-year-old man presenting with fever, myalgia, anosmia, and ageusia, tested positive for SARS-CoV-2 on pharyngeal swab, yet also tested negative for viral RNA in semen and urine 8 days later. Furthermore, in a study that investigated testicular samples on autopsy, Flaifel et al. [42] (2021) reported no evidence of viral RNA in testes of 10 patients who had succumbed to COVID-19 while they remained positive for nasopharyngeal tests. This is supported by a post-mortem investigation of a 67-year-old male, reporting a negative test on the testis samples studied [36].

## SARS-CoV-2 and semen quality

Two studies were found that primarily investigated the impact of SARS-CoV-2 infection on semen quality, reporting a negative impact on sperm quality (Table 2). Li et al. [43] (2020) investigated 23 patients recovering from COVID-19 infection in a hospital-based case-controlled cross-sectional study. This study reported that patients recovering from COVID-19 had significantly decreased sperm concentration and increased seminal interleukin 6 (IL-6), TNF-α, and monocyte chemoattractant protein-1 (MCP-1) compared to control. Furthermore, 39.1% patients had oligozoospermia and 60.9% patients had increased leucocytes. Immune orchitis was also reported in some patients in this cohort. Maleki et al. [44] (2021) investigated 84 patients recovering from COVID-19 in a case-controlled longitudinal study compared to 105 healthy controls, with evaluations at 10-day intervals over a maximum follow-up of 60 days. A significant reduction in semen volume, sperm concentration, progressive motility, and sperm morphology were reported. This was associated with increased ACE2 enzyme activity in seminal plasma, increased seminal inflammation (IL-1β, IL-6, IL-8, IL-10, TGF-β, TNF-α, interferon α [IFN-α], IFN-γ), oxidative stress (increased reactive oxygen species [ROS] and reduced superoxide dismutase [SOD]) and increased seminal apoptotic markers activity (caspase-8, −9, and −3) compared to control.

An additional five studies included under the presence of viral RNA in semen also reported semen analysis results. In a case-controlled cohort study that included 16 patients recovering from COVID-19 (mean [SD] age 42.2 [9.9] years), two patients with acute COVID-19 and 14 control patients (mean [SD] age 33.4 [13.1] years) that were negative for SARS-CoV-2, Holtmann et al. [37] (2020) reported that moderate COVID-19 infection significantly reduced sperm concentration, progressive motility and total number of complete motility compared to men recovered from a mild infection and the control group. Semen parameters were found to be impacted in a case-controlled study of 74 men (median age 34 years) recovering from a confirmed infection, where 14.9% were asymptomatic (mild), 41.9% were classified as moderate, and 43.2% had severe pneumonia. Sperm concentration, total sperm count, and total motility were affected, but not volume or progressive motility. Only sperm concentration showed a correlation with disease severity, and longer recovery times (>90 days) also had significantly lower sperm concentration that shorter recovery (<90 days) [33]. Of 23 men (age range 20–62 years) in acute or convalescence stage of COVID-19, all patients had total sperm count, total motility and morphology within normal parameters [40]. In a case-controlled cross-sectional study that included confirmed and suspected COVID-19 cases pre-treatment (*n*= 10) and post-treatment (*n*= 10) to a healthy control group (*n*= 10), no significant differences in semen parameters were reported. However, normal sperm morphology was found to be reduced in the pre-treatment group compared to control, but this was not observed post-treatment [35]. Gacci et al. [29] (2021) reported in a cross-sectional cohort of sexually active men that were confirmed to be recovered from COVID-19 (*n*= 43) that 25% where found to be oligo-crypto-azoospermic, and this was related to severity of COVID-19 infections, exceeding the rates of found in the average population. Additionally, this study reported increased seminal IL-6 in 76% of the cohort, further suggesting that reproductive tract inflammation may be associated with COVID-19 infections in males. Furthermore, Ma et al. [30] (2021) reported a cohort of 12 COVID-19 male patients, where 66.7% of the patients had normal sperm parameters and low sperm DNA fragmentation (SDF), whereas 33.3% of the patients had low sperm motility with higher SDF.

## SARS-CoV-2 on testicular histology

The present review identified five studies that reported testicular histology or US outcomes in patients infected with COVID-19 (Table 3).

Flaifel et al. [42] (2021) investigated testes and epididymis specimens from 10 patients (age range 23–83 years) who had succumbed to COVID-19 following positive nasopharyngeal swabs for viral RNA at time of admission to hospital. Each patient had one or more comorbidities, including type 2 diabetes mellitus and hypertension, and remained positive for viral RNA in the nasopharyngeal tract but not the testicular samples at autopsy. The authors found that seven COVID-19 samples had morphological changes consistent with oxidative stress, including altered chromatin condensation, acidophilic cytoplasm, and DNA fragmentation. Spermatocytes were also found to be sloughed into the tubules in the epididymis. Furthermore, the Sertoli cells showed cellular swelling and vacuolisation. In cases that had a longer duration, hypertrophy of the tubular basement membrane and reduced intratubular cell mass was reported. Interestingly, two cases also showed signs of multiple microthrombi, associated with increased platelets in the testicular tissues. Only one case showed signs compatible with orchitis, namely influx of cluster of differentiation 8 (CD8)-positive dominated mononuclear leucocytes into the interstitial space.

Ma et al. [45] (2021) examined testicular tissue in five males who had died from COVID-19 complications (age range 51–83 years) and compared these to non-COVID-19 (age range 71–80 years) affected testicular samples. All five COVID-19 patients all showed degenerated germ cells (GCs) sloughed off in the seminiferous tubules, whereas the controls showed normal GC development within the seminiferous tubules. They observed that two patients showed almost no GCs, appearing similarly to Sertoli cell-only syndrome. The patients with COVID-19 also showed reduced DDX4-positive GC needed for regulation of GC proliferation and differentiation. However, there was no difference found with Sertoli cells between patients and controls. SARS-CoV-2 may therefore impair GC development, but not affect the Sertoli cells.

Achua et al. [31] (2021) analysed testicular specimens on autopsy from six COVID-19-positive males (age range 20–87 years) compared to three COVID-19-negative controls (age range 28–77 years), with all patients having relevant comorbidities. It was observed that three of the COVID-19 cases showed impaired spermatogenesis. Only one case reported infiltration of macrophages and lymphocytes within the testicular tissues. All other cases did not show any signs of inflammation. Interestingly, in the three patients with COVID-19 with normal spermatogenesis, ACE2 receptor expressions was significantly lower compared to the patients with COVID-19 with impaired spermatogenesis. The patients with impaired spermatogenesis and reduced ACE2 receptor expression also showed Sertoli cell-only syndrome, early maturation arrest, and sclerosis of the seminiferous tubules.

Yang et al. [46] (2020) analysed 11 patients’ testes on autopsy who had died from COVID-19 (age range 42–87 years), where 10 of these patients had a history of fever, and 10 received low-dose steroidal therapy. This was compared to five uninfected control autopsy cases negative, for COVID-19 Sertoli cells were most notably affected by ‘ballooning’ morphological changes, with swelling and hydrolysation alongside detachment from the basement membrane. These findings were classified as 18.2%, 45.5%, and 36.4% of 11 cases showing mild, moderate, and several injuries, respectively, compared to controls that showed no injuries or mild tubular derangements only. In addition, Leydig cell counts in COVID-19 cases was significantly reduced compared to the controls. Furthermore, there was oedema and mild inflammatory cellular influx.

Chen et al. [47] (2020), in a retrospective study of a cohort of 142 confirmed COVID-19-positive patients (age range 24–93 years), examined US reports made at the bedside. These cases were classified as mild or moderate (41.9%) and severe or critical (58.5%), with the latter group significantly older and more likely to have coronary heart disease or chronic obstructive pulmonary disease compared to the mild/moderate group. Acute orchitis was present in 10% of all patients, with 7% presenting with acute epididymitis, and 15% presenting with acute epididymo-orchitis. Additional manifestations observed in all patients was a thickened tunica albuginea (25.4%), increased testicular vascular flow (20,4%), increased epididymal vascular flow (16,9%), heterogeneous echogenicity of testis (9.9%), scrotal swelling (8.5%), enlargement of epididymis (7.7%), hydrocele (7,7%), enlargement of testis (7%), heterogeneous echogenicity of epididymis (5.6%), and abscesses in the epididymis (2.8%). Severe and critical cases had significantly increased percentage of patients with thickened tunica albuginea, heterogeneous echogenicity of the testis, increased testicular and epididymal vascular flow and absence in the epididymis compared to mild and moderate cases. Importantly, there was an increased risk of scrotal infection within this age group of the cohort.

Additional information is reported in a study categorised under semen quality, where Li et al. [43] (2020) reported testicular damage in six age-matched case-controlled autopsies on testicular tissue. This included inflammatory infiltration into testicular and epididymal tissue, signs of oedema, congestion and red blood cell exudates. The seminiferous tubules were thinned with an increased number of apoptotic cells were present, as well as increased CD3^+^ and CD68^+^ in the interstitial cells of testicular tissue and the presence of immunoglobulin G (IgG) within seminiferous tubules of autopsied testes. Furthermore, Song et al. [36] (2020) reported that there was no detection of SARS-CoV-2 viral RNA in the testis of an autopsied 67-year-old male who had succumbed to COVID-19.

## SARS-CoV-2 on testicular clinical presentations

There were two studies primarily reporting testicular clinical symptoms in COVID-19 infections (Table 4). Patients aged between 18 and 75 years diagnosed with COVID-19 (*n*= 91) were evaluated for testicular pain or epididymo-orchitis. Here, 11% of patients presented with pain, and only one was clinically diagnosed with epididymo-orchitis. Subsequent comparison of patients with the presence (*n* = 10) or absence (*n* = 81) of testicular pain showed no difference for neutrophil count, lymphocyte count, C-reactive protein (CRP), D-dimers or duration of COVID-19 infection between these groups, where lymphopenia was significantly more common in older patients, but not testicular pain or swelling [48]. Kim et al. [49] (2020) reported the case of 42-year-old adult male presenting in the emergency room with severe abdominal pain and testicular pain, without respiratory symptoms, that was subsequently diagnosed with COVID-19. In a cohort of 34 male patients recovering from COVID-19, Pan et al. [34] (2020) reported a prevalence of 19% for symptoms indicative of orchitis.

## SARS-CoV-2 infection and male reproductive hormones

There were two studies primarily reporting reproductive outcomes in COVID-19 infections (Table 5). Kadihasanoglu et al. [50] (2020) conducted a case-controlled cross-sectional study on 89 patients classified as hospitalised for confirmed COVID-19 (mean [SD] age 49.9 [12.5] years), 30 hospitalised cases with non–COVID-19 (two or more negative tests) respiratory tract infection (mean [SD] 52.7 [9.6] years) and 143 age-matched controls (mean [SD] 50 [7.8] years). The COVID-19 cohort was described as mild (52.8%), moderate (33.7%) and severe (13.5%). Testosterone was significantly lower, and LH and prolactin were higher in the COVID-19 cohort compared to non-COVID-19 infections and control groups, with no differences between groups for FSH. Furthermore, patients with COVID-19 reported significantly lower white blood cell count (WBC) and lymphocyte count and higher CRP compared to controls, where WBC and lymphocytes, but not CRP, were significantly increased in patients with COVID-19 compared to non-COVID-19 patients. These variables were found to be associated with the severity of disease and were further significantly worse in patients classified as severe COVID-19. Testosterone also negatively correlated with hospital duration and positively correlated with oxygen saturation in patients with COVID-19, but not in the non-COVID-19 infection group. No correlations were found for testosterone compared to neutrophil count, lymphocyte count or CRP. In four patients who succumbed to COVID-19, the testosterone levels of these cases was below the median for the COVID-19 cohort [50].

Ma et al. [30] (2021) investigated 119 male patients (age range 20–49 years) for reproductive hormones, where all patients had a stable clinical status during the study in the hospital setting. This was compared to 273 age-matched controls (age range 24–49 years) who were undergoing reproductive hormonal analysis for fertility prior to marriage or planning parenthood. The COVID-19 cohort was described as mild (84.0%), moderate (11.8%) and severe (1.96%), and case management included use of corticosterone (14.8%), arbidol (45.3%), oseltamivir (33.6%), or intravenous antibiotics (56.3%). In 39% of the cases liver function (elevated alanine aminotransferase [ALT] and aspartate aminotransferase [AST]) was impaired. The reproductive hormonal analysis showed that LH was significantly higher in the COVID-19 cohort compared to the control, whereas no difference in testosterone, FSH and oestrogen levels were found between groups.

Okçelik et al. [51] (2020) studied 44 patients (mean[SD] age 35.5 [9.9} years) in a COVID-19 outpatient clinic. Testosterone, LH and FSH were not significantly different in patients who tested positive for COVID-19 (*n* = 24) compared to negative patients (*n* = 20). In 42 patients that had a chest CT scan, COVID-19 pneumonia was diagnosed in 23. Testosterone was significantly lower in the pneumonia group compared to those without pneumonia.

Rastrelli et al. [52] (2020) investigated 31 patients recovering from SARS-CoV-2 pneumonia in the respiratory intensive care unit of Carlo Poma Hospital, Mantua, Italy. It was found that 67.7% improved in clinical presentation (age range 55–66 years), 19.4% remained stable (age range 33–83 years) and 12.9% worsened or died (age range 59–85 years) at time of analysis. There was a significantly lower total and free testosterone and higher LH in the severe/deceased group compared to the other groups. These patients also reported significantly increased CRP, procalcitonin and neutrophil count with lower lymphocytes. The authors concluded that lower baseline levels of testosterone predict mortality outcomes in patients with SARS-CoV-2 infections that are in intensive care units.

## Discussion

Initial fears of a negative impact of SARS-CoV-2 infection on male fertility resulted in many couples delaying pregnancy and fertility treatment, although many have now resumed their pursuit of parenthood and are accepting the uncertainty [22]. Guidelines for assisted reproductive technology (ART) management have also been issued by professional societies such as the American Society for Reproductive Medicine (ASRM), European Society of Human Reproduction and Embryology (ESHRE) and International Federation of Fertility Societies (IFFS) [53], with concerns raised on impact and transmission of the virus on fertility and ART [54,55]. This has included recommendation for gonadal function assessment and semen analysis in males of reproductive age who have recovered from COVID-19 [56]. There is a further concern that SARS-CoV-2 may be transmitted sexually, or via ART [22,23,25], which requires the presence of the virus in seminal or vaginal fluids for such a mode of transmission [57].

SARS-CoV-2 gains access to cells via ACE2 receptor binding with TMPRSS2 priming [7,8]. The testes are reported to express ACE2, particularly in the spermatogonia, Leydig cells and Sertoli cells, which leads to the hypothesis of SARS-CoV-2 gaining access to testicular tissues and seminal fluid, directly affecting male reproductive health [58]. TMPRSS2 has been reported to be present in prostate epithelial cells, where the expression is regulated by androgens, and present in semen as components of prostasomes [59,60]. These proteasomes or the ‘exosome-like structures’ reportedly may incorporate TMPRSS2 in the sperm membrane [60]. Therefore, although ACE2 is expressed in male reproductive tissues, there is generally a lack of TMPRSS2 modulatory protein co-expression except in prostatic epithelial cells [57,59], and the presence of ACE2 and TMPRSS2 together in male reproductive tissues is considered relatively low [24].

Although ACE2 receptors are found to be expressed in male reproductive tissue, specifically the testis [58], and TMPRSS2 has been reported to be present in prostate epithelial cells and in semen [59,60], it is still not clear whether SARS-CoV-2 does gain access directly to the male reproductive tract. In an early systematic review, Khalili et al. [27] (2020) reported that the virus may be present in seminal fluid, with limited evidence of reduced semen parameters and testosterone. Yao et al. [61] (2021) concluded that there is limited evidence for the virus in seminal fluid; however, fatal cases appear to have damaged testicular structures in the absence of the virus. A subsequent systematic review investigating viral presence of SARS-CoV-2 in male and female reproductive tracts concluded that the virus is unlikely to be considered a sexually transmitted virus. This included semen, testicular biopsies, prostatic fluid, vaginal fluids, and oocyte samples. However, the induction of orchitis, or having an impact on spermatogenesis and semen analysis findings or testosterone level is still possible [57]. These results are generally consistent with the results of this systematic review, where there is little evidence suggesting viral presence in the seminal fluid or reproductive tract. There is also little evidence to suggest SARS-CoV-1 is present in seminal fluid [62–64]. Therefore, the evidence for sexual transmission is also currently lacking, particularly as the presence of viral nucleic acids in seminal fluid does not equate to an infectious intact virus [64].

Although there have been 27 viruses reported to gain access to semen through viraemia, including Hepatitis B and C, Adenovirus, HIV, Mumps, Ebola, Zika and numerous herpes family viruses reported in seminal fluid [62–64], the seemingly low risk of SARS-CoV-2 presence in seminal fluid may be due to the low levels of both ACE2 and TMPRSS2 expressed in the testis [24]. Pan et al. [34] (2020) suggested that ACE2 mediated viral entry into target cells is unlikely to occur within the human testis as single-cell transcriptome analysis demonstrates sparse expression of ACE2 and TMPRSS2. Further evidence supporting this is from a retrospective cohort study using cryopreserved semen samples obtained from young healthy adult sperm donors at the Hunan Province Human Sperm Bank (China), where paired blood and semen samples from 50 donors during the first wave of the pandemic, and 50 donors collected upon work resumption, where analysed. This study reported that all samples from during and after the pandemic wave were free of SARS-CoV-2 detection and safe for external use [65].

The 27 viruses detected in human semen all come from diverse families, suggesting that mechanisms for viral access may not be unique to specific viral epitopes. Non-specific mechanisms could be through serum viral load, inflammatory mediators altering the blood–testis barrier (BTB), testicular immunosuppression that may protect viruses from testicular immune surveillance, and pyrexia [54,62,64,66]. The few accounts of the presence of SARS-CoV-2 may be through a BTB breach, and may account for subsequent damage to GCs and testicular interstitial tissues [24]. However, there are still too few studies for appropriate conclusions in COVID-19, and these studies currently have small sample sizes, and mostly confined to men with mild-to-moderate symptoms.

Reproductive tract inflammation and infections are causes of male infertility, most prominently bacterial infections, environmental causes and autoimmunity [66]. This may also include systemic autoimmune disease and chronic asymptomatic inflammation associated with obesity, metabolic syndrome and diabetes mellitus [67,68]. It is known that viral infections may negatively affect fertility, including adenovirus, herpes simplex virus, HIV, hepatitis B and C [63,69–71]. More recently, in was reported that the Zika virus may also alter semen parameters and reduce male fertility [63,64]. Although there remains a paucity of studies, there is current evidence for impaired sperm parameters in COVID-19, mostly in males recovering from infection. This is consistent with the review conducted by Meng et al. [72] (2020), suggesting that SARS-CoV-2 can cause spermatogenesis dysfunction and impaired sperm parameters. However, as there is little evidence suggesting the presence of the virus in reproductive tissues, this is most likely mediated by non-specific mechanisms. The viral-induced damage is proposed through changes to testicular structure and/or function, immune cell infiltration into the testicular compartment, a systemic inflammatory response, pyrexia, reduced testosterone, and potential incorporation of the virus genetic material into the GC genome [63,66,69].

Although inflammatory cytokines such as TNF-α, IL-1β, and IL-6 have physiological functions, increased levels with inflammation can negatively impact spermatogenesis. Inflammation is also typically associated with oxidative stress [67,73]. Males are also commonly found to have acute or chronic inflammation of the reproductive tract in an infertility assessment [67,73]. Effects of acute febrile illness induced in rats showed impaired spermatogenesis and increased GC apoptosis that was not consistent with hormonal changes, suggesting direct impact of the immune response [74]. GCs have also been shown to have receptors for TNF, IL-1 and IL-6, supporting a direct action of these cytokines in the induction of apoptosis [75]. Furthermore, febrile illness due to viral infection, including influenza and SARS-CoV-1, is known to negatively impact sperm parameters, including concentration, motility, and morphology [63]. Based on the limited evidence available, it is likely that inflammation and oxidative stress associated with COVID-19 may negatively impact spermatogenesis. This is supported by histological studies suggesting inflammatory and oxidative stress mediated damage. Therefore, clinical investigation for males recovering from COVID-19 may be warranted, as well as consideration in the clinical assessment of infertile males. Furthermore, the current evidence may warrant the consideration of cryopreservation in ART patients at high risk of SARS-CoV-2 exposure or a severe clinical course of COVID-19, particularly as long-term reversibility is not yet established.

Numerous viruses that cause viraemia have been associated with orchitis, including influenza, Coxsackie B, SARS-CoV-1, rubella, smallpox, echovirus, and parvovirus [62–64]. Mumps orchitis is arguably the most well-known viral disease that can be complicated with orchitis in males, and may cause testicular atrophy and infertility [76]. Furthermore, orchitis was found to be a complication of SARS-CoV-1 viral infections [77]. The limited results of the present review suggest that orchitis is a possible complication in COVID-19. Viral orchitis is associated with infiltration of neutrophils, macrophages and T and B lymphocytes, with degeneration of germinal epithelium and few to no spermatogonia and Sertoli cells, lamina propria hypertrophy and fibrosis of the tubules. However, Leydig cells generally do not show signs of cellular injury [66]. Orchitis and histological damage to testicular tissues in COVID-19 may further be mediated by a vasculitis, as hypercoagulability and the segmental vascularisation structure of the testis may induce inflammation [56]. This is consistent with evidence of testicular autopsy in patients with severe phase 2 infection who ultimately died. This was also reported for SARS-CoV-1 autopsy specimens of patients (*n*= 6) that had died, where the testes also showed GC destruction, few spermatozoa in the seminiferous tubules, basement membrane hypertrophy, leucocyte infiltration (CD3^+^ T lymphocytes and CD68^+^ macrophages) and IgG precipitation in seminiferous epithelium. This was also without detection of SARS-CoV-1 genomic sequences in the samples, further supporting immune-mediated damage to testicular tissues [77].

Although there are studies suggesting female predominance or no sex disparity, there is increasing evidence of a male predominance for COVID-19 morbidity and mortality [10,12]. In a meta-analysis, Peckham et al. [13] (2020) reported no difference between male and female gender for confirmed infections, where males were reported to have a 2.84 increased risk of intensive care treatment and 1.39 increased risk of death. Similar increased sex differences in COVID-19 fatality has been reported in data from Europe, China and Korea [14–17]. Moreover, observational studies further report that the severity of COVID-19 intensive care and mortality rates are increased in males [18,19]. This has also been found to be similar regarding retrospective data that was retrieved from patients with SARS-CoV-1 [16]. Males may also shed the SARS-CoV-2 virus for longer duration compared to females [78]. This has led to the suggestion that androgens may be involved in the pathogenesis of COVID-19.

Testosterone is known to be reduced in comorbidities associated with COVID-19, including ageing, obesity, diabetes and chronic obstructive pulmonary disease [79]. Hypogonadism is further associated with increased inflammatory cytokines, and testosterone reduces IL-1β, IL-6, and TNF-α, which is accentuated in ageing. Although only four studies are included that investigated androgens in patients with COVID-19, the results of this review suggest that testosterone may be reduced in male patients with COVID-19 compared to controls, and this is negatively associated with CRP as a systematic marker of inflammation. Furthermore, in a non-peer reviewed published article, Schroeder et al. [80] (2020) reported that 68.6% of males with COVID-19 in intensive care units (Hamburg, Germany) had lower serum testosterone levels and 48.6% had lower dihydrotestosterone levels, which was negatively correlated with severity and death. Lower testosterone was negatively correlated with IL-2 and INF-γ, and oestradiol positively correlated with IL-6, as did disease severity. Reduced testosterone may therefore be involved in the pathogenesis of the cytokine storm and complications of phase 2 COVID-19 [79]. This reduced testosterone may further weaken respiratory muscles and reduce lung function parameters such as forced expiratory volume [79]. However, female patients may be generally less susceptible to viral infections through a better immune response due to the activity of oestrogen in activating T cells, a reduced expression of inflammatory cytokines, and increasing antibody formation in B-lymphocyte humoral immune response [12]. Male patients also have higher expression of ACE2 in the renal system compared to females, which may further explain a gender disparity [12].

## Conclusion

There is little evidence suggesting SARS-CoV-2 viral presence in the male reproductive tract and that sexual transmission may occur. However, COVID-19 infections may have a negative impact on spermatogenesis and male fertility. The current evidence available suggests that non-specific mechanisms associated with the systemic and local reproductive immune response to the SARS-CoV-2 virus could explain the impact. This may also be associated with orchitis and associated testicular ultrasonography changes. COVID-19 may further decrease testosterone, which in turn exacerbates the inflammatory response. The clinical evaluation of male reproductive tract, seminal parameters and reproductive hormones are recommended in men undergoing fertility treatment. Any long-term negative impact on male reproduction remains unexplored and requires further future consideration.
